# Dedifferentiated Leiomyosarcoma of the Uterine Corpus with Heterologous Component: Clinicopathological Analysis of Five Consecutive Cases from a Single Institution and Comprehensive Literature Review

**DOI:** 10.3390/diagnostics14020160

**Published:** 2024-01-10

**Authors:** Suyeon Kim, Hyunsik Bae, Hyun-Soo Kim

**Affiliations:** 1 Department of Pathology and Translational Genomics, Samsung Medical Center, Sungkyunkwan University School of Medicine, Seoul 06351, Republic of Korea; ssuuyy.kim@samsung.com; 2 Pathology Center, Seegene Medical Foundation, Seoul 04805, Republic of Korea

**Keywords:** uterus, leiomyosarcoma, dedifferentiated, heterologous component, chondrosarcoma, rhabdomyosarcoma

## Abstract

Dedifferentiation is a very rare phenomenon in uterine leiomyosarcoma (LMS). The aim of this study was to comprehensively analyze the clinicopathological characteristics of uterine dedifferentiated LMS (DDLMS). We reviewed electronic medical records and pathology slides from five patients with uterine DDLMS and performed immunostaining. The mean age of the patients was 56 years. Two patients presented with abdominal discomfort, while in three cases the uterine tumors were detected on routine medical examination. The mean size of the tumors was 17.0 cm. Four patients underwent hysterectomy. The initial stages were distributed as IB (2/5), IIIC (2/5), and IVC (1/5). Post-operative concurrent chemoradiation therapy, radiation therapy, and chemotherapy were administered in one, one, and two patients, respectively. Despite post-operative treatment, three patients developed metastatic recurrences in the abdominal and pelvic organs. Recurrence-free survival time ranged between 4 and 30 months. Histologically, the differentiated areas demonstrated the classic morphology of malignant smooth muscle differentiation, whereas the dedifferentiated areas resembled undifferentiated pleomorphic sarcoma and were characterized by large pleomorphic tumor cells admixed with haphazardly arranged atypical cells with marked nuclear pleomorphism. All cases also exhibited heterologous components, including chondrosarcoma (CSA; 3/5) and rhabdomyosarcoma (2/5). In two cases, the heterologous components were initially detected in primary tumors. In three cases, the primary tumors did not exhibit any dedifferentiated or heterologous components. Instead, more than half of the recurrent tumors consisted of heterologous components. Three cases showed a sharp demarcation between the LMS and CSA components, while in two cases the dedifferentiated area imperceptibly merged with the differentiated component. Immunostaining revealed that the dedifferentiated components exhibited a lack of desmin immunoreactivity in three of the four examined cases. A subset of uterine LMS represents various amounts and types of dedifferentiation and heterologous components in both primary and recurrent tumors. Routine recognition of DDLMS and distinction from its mimickers are required for accurate diagnosis and further characterization of these rare tumors.

## 1. Introduction

Leiomyosarcoma (LMS) is a mesenchymal malignancy that primarily develops from smooth muscle cells in both visceral organs and non-visceral structures [1,2]. It is the most common malignant soft tissue tumor, comprising 15% of all sarcomas in adults [3]. The most common primary sites in which LMS develops include the uterus, retroperitoneum, gastrointestinal tract, and extremities, although its distribution is broad [2,4]. Its clinical behavior has a wide range of outcomes, primarily based on histological grade, stage, and distant metastasis [5].

Uterine sarcoma is one of the rarest female genital tumors, representing only 1% of all gynecological malignancies [6,7]. Arising from the myometrium or the connective tissue elements of the endometrium, uterine sarcoma includes a heterogeneous group of tumors derived from mesenchymal cells, comprising three main histological types: LMS, endometrial stromal sarcoma, and undifferentiated uterine sarcoma [8]. Uterine LMS constitutes 50–70% of uterine mesenchymal malignancies, with an annual incidence of approximately 1 per 100,000 women in United States [9,10]. The majority of uterine LMS is identified in perimenopausal women aged 50–55 years, although 15% is found in women aged <40 years [11,12]. It is more prevalent in younger women, with increasing incidence at 30 years and a peak at 50 years [2]. Uterine LMS is often aggressive, with a worse prognosis than endometrial endometrioid carcinoma. Typically presenting as a large pelvic mass, its signs and symptoms include vaginal bleeding, pelvic pain, a sense of fullness in the pelvis or abdomen, dyspareunia, and dysuria, although some patients are asymptomatic. Since uterine LMS is rarely suspected before surgery, this tumor is often diagnosed upon routine pathological examination after hysterectomy or myomectomy performed to treat presumed uterine leiomyoma [13,14].

Dedifferentiation has been documented in several malignant mesenchymal tumors, such as liposarcoma, chondrosarcoma (CSA), rhabdomyosarcoma (RMS), chordoma, and solitary fibrous tumor [15]. These dedifferentiated sarcomas are more aggressive than those without the dedifferentiated component [16]. Histologically, dedifferentiation is defined as an abrupt transition from the differentiated area to the undifferentiated tumor [3]. The term dedifferentiated LMS (DDLMS) was first coined by Shmookler and Lauer in 1983 [17], who determined that DDLMS is morphologically characterized by the abrupt transition from classic LMS to high-grade undifferentiated pleomorphic sarcoma (UPS). The latter does not express immunohistochemical smooth muscle markers [18,19].

Although a small number of DDLMS cases has been reported in the retroperitoneum, trunk, and extremities [20,21], uterine DDLMS is relatively rare [22]. Its clinical features, pathological characteristics, and immunophenotypes have yet to be clarified. Previous studies have shown that, in some uterine DDLMS cases, the non-myogenic tumor component presents heterologous differentiation. The heterologous components, such as RMS, CSA, and osteosarcoma, frequently display severe nuclear pleomorphism, while those exhibiting low-grade cytological atypia have seldom been reported [23]. We recently experienced some cases of primary uterine DDLMS with a heterologous component and initiated a comprehensive review of previously published cases with thorough analysis of their clinicopathological characteristics. In this study, we investigated the clinical manifestations, histological features, and immunostaining results of uterine DDLMS. Our comprehensive analysis will improve the understanding of this rare condition and help pathologists to make accurate diagnoses.

## 2. Materials and Methods

With the approval of the Institutional Review Board at the Samsung Medical Center (Seoul, Republic of Korea), the pathology database was queried for all cases of primary uterine LMS between January 2021 and December 2023. During the 3 years of the study period, a total of 103 patients in the database underwent surgery for primary uterine LMS. Two board-certified gynecological pathologists examined all available hematoxylin and eosin (H&E)-stained slides to confirm the diagnosis and select the most representative formalin-fixed, paraffin-embedded (FFPE) tissue blocks for immunohistochemical staining. Upon a thorough slide review, we identified five patients with primary uterine DDLMS and reviewed their electronic medical records to collect the following clinical information: patient’s age at initial diagnosis; presenting symptoms; previous medical history; imaging findings; pre-operative clinical impression; pathological diagnosis; greatest dimension of uterine tumor; surgical procedure for uterine tumor; extension to the adnexa, pelvic peritoneum, abdominal peritoneum, rectum, and bladder; lymph node metastasis; initial stage; post-operative treatment and recurrence; pathological diagnosis of recurrent tumor; treatment for recurrence; recurrence-free survival; survival status; and overall survival. We also reviewed all available H&E-stained slides to analyze the following pathological characteristics: dominant morphology of the differentiated component; nuclear pleomorphism; mitotic count per 10 high-power fields; tumor cell necrosis; tumor border; intratumoral lymphocytic infiltrate; and histological type and proportion of the dedifferentiated component.

Immunostaining was performed using whole-tissue sections containing >80% viable tumor tissue, as previously described [24,25,26,27,28,29,30,31,32]. In brief, 4 μm thick FFPE tissue sections were deparaffinized and rehydrated using a xylene and alcohol solution. We used an automated instrument (BOND-MAX immunostainer; Leica Biosystems, Deer Park, IL, USA) with a biotin-free polymeric horseradish peroxidase-linker antibody conjugate system (BOND Polymer Intense Detection System; Leica Biosystems) [33,34,35,36]. After antigen retrieval, endogenous peroxidases were quenched with hydrogen peroxide. The sections were incubated with the primary antibodies listed in Table 1. After chromogenic visualization, the sections were counterstained with hematoxylin and coverslipped. The appropriate positive controls listed in Table 1 were stained, while the negative control was prepared by substituting non-immune serum for the primary antibodies, resulting in no detectable staining. For desmin and S100 protein, staining in the cytoplasm or membrane was interpreted as positive expression. For myogenin, myoD1, and special AT-rich sequence-binding protein 2 (SATB2), nuclear staining was interpreted as positive expression [24,28,37]. The staining intensity was graded as strong, moderate, or weak, while the staining proportion was graded as either diffuse (≥50%) or focal (<50%). For retinoblastoma protein (RB), even weak nuclear staining indicated preserved expression, whereas loss of expression was defined as the complete absence of nuclear immunoreactivity. p53 expression was interpreted as a mutation pattern when one of the following staining patterns was observed [38]: diffuse (≥75%) and strong nuclear immunoreactivity (overexpression); no nuclear immunoreactivity in any of the tumor cells (complete absence); or unequivocal cytoplasmic staining (cytoplasmic). In contrast, p53 expression was interpreted as a wild-type pattern if a variable proportion of nuclear expression with mild-to-moderate staining intensity was observed.

## 3. Results

**Figure 1:**
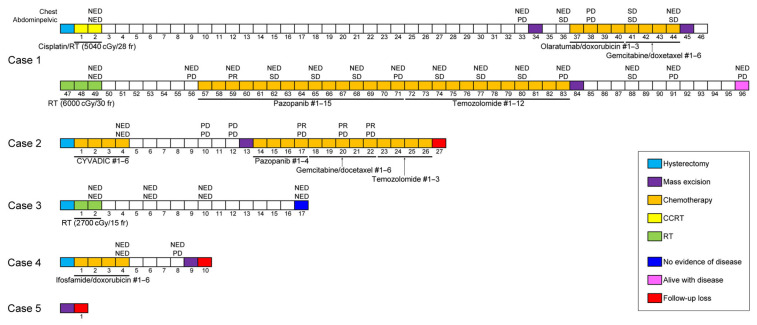
Timelines of patients’ clinical course. #—cycle of chemotherapy; CCRT—concurrent chemoradiation therapy; NED—no evidence of disease; PD—progressive disease; PR—partial response; RT—radiation therapy; SD—stable disease.

Figure 1 summarizes the patients’ treatment timelines. In case 1, the patient underwent hysterectomy and adjuvant concurrent chemoradiation therapy (CCRT). Thirty-three months after surgery, she developed a recurrent tumor in the pelvic peritoneum and underwent repeated surgeries and radiation therapy to treat the peritoneal metastatic recurrence. After 7 disease-free months, the abdominal metastases progressed again. The patient then underwent surgical excision and received chemotherapy for 26 months. She has survived with this disease for 96 months after a hysterectomy. In case 2, the patient underwent hysterectomy with post-operative chemotherapy. The pulmonary and peritoneal metastases developed 10 months after the hysterectomy. Surgical mass excision was performed, and chemotherapy was administered for 12 months. In case 3, the patient was prescribed post-operative radiation therapy, but the treatment was interrupted due to exacerbation of her health condition. Nevertheless, 17 months after the operation, she is alive with no signs of recurrent disease. In case 4, the patient underwent hysterectomy with adjuvant chemotherapy but developed a recurrent tumor in the left paracolic gutter 8 months later. She then underwent surgical excision. In case 5, the patient with advanced-stage disease was lost to follow-up immediately after extensive surgery.

Table 2 summarizes the clinical features of patients with uterine DDLMS. Patient age ranged between 51 and 63 years (mean, 56 years). Two patients presented with abdominal discomfort (cases 1 and 5), one with constipation (case 1), and another with a palpable pelvic mass (case 5), while in the remaining three asymptomatic patients the uterine tumors were detected during routine medical examinations. Ultrasonography revealed single (cases 2 and 4) or multiple (case 3) uterine masses occupying the abdominal or pelvic cavity. Two patients had previous medical histories of thyroid carcinoma and umbilical hernia (case 3) and hyperthyroidism (case 4), respectively. Imaging findings were available for four patients. Abdominopelvic magnetic resonance imaging revealed various radiological features, including a mixed solid and cystic mass (case 2), multiple solid masses (case 3), a heterogeneous mass with intratumoral necrosis (case 4), and adnexal extension (case 5). Four patients were suspected of having uterine leiomyoma, while the remaining patient (case 5) was presumed to have uterine sarcoma. Four patients underwent total hysterectomy. One and two patients underwent bilateral salpingectomy (case 3) and salpingo-oophorectomy (cases 2 and 4), respectively. In one patient (case 2), pelvic and para-aortic lymph node dissection was performed. The remaining patient (case 5), who did not undergo hysterectomy, received uterine mass excision with left oophorectomy, left hemicolectomy, and left nephrectomy. Two patients (cases 2 and 5) were initially diagnosed with primary uterine DDLMS with a heterologous component, while in the remaining cases, the uterine tumors were diagnosed as LMS (cases 1 and 3) and DDLMS (case 2), respectively. The dimensions of the uterine tumors ranged between 8.3 and 27.8 cm (mean, 17.0 cm). The initial stages were distributed as IB (2/5; cases 1 and 2), IIIC (2/5; cases 3 and 4), and IVC (1/5; case 5). Lymph node metastases were detected in two patients (cases 3 and 4), while pelvic and abdominal peritoneal metastases were identified in one (case 3) and two (cases 3 and 5) cases, respectively.

Follow-up information was available for all but one patient (case 5; Table 2). Post-operative chemotherapy, CCRT, and radiation therapy were administered to two (cases 2 and 4), one (case 1), and one (case 3) patients, respectively. The latter patient was initially prescribed radiation therapy for the whole pelvis and para-aortic area (at an intended dose of 5400 cGy in 30 fractions), but the treatment was interrupted after receiving 2700 cGy when the patient’s health worsened. Two patients received six cycles of post-operative chemotherapy, including cyclophosphamide, vincristine, adriamycin, and dacarbazine (case 2), and ifosfamide and doxorubicin (case 4), respectively. One patient (case 1) received post-operative radiation therapy (5040 cGy in 28 fractions) with six cycles of weekly cisplatin. Despite the post-operative treatment, three of the four patients (cases 1, 2, and 4) developed metastatic recurrences in the abdominal and pelvic organs, including the vagina, ovary, bladder, colon, mesentery, omentum, and abdominopelvic peritoneum. Four metastatic tumors had heterologous components. One patient (case 3) did not experience any recurrent or metastatic disease during 15 months after hysterectomy; however, one of the patients (case 2) experienced distant metastases in the lungs, and three patients underwent surgical excision for metastatic tumors. Two patients also received CCRT (case 1) and chemotherapy (case 2), respectively. The recurrence-free survival time ranged between 4 and 30 months (mean, 13.3 months). One of the three patients who developed recurrences is currently alive with disease.

**Figure 2:**
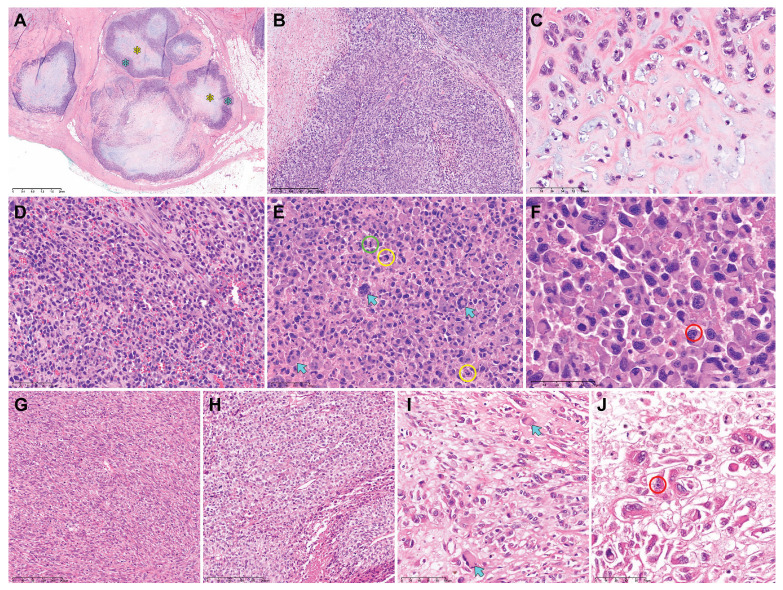
Histological features of uterine DDLMS (cases 1−3). Case 1: (**A**) The metastatic lesion exhibits variable-sized multinodular tumors. Each nodule consists of a peripheral rim of dedifferentiated component (blue asterisks) and CSA (yellow asterisk) at the center. (**B**) The dedifferentiated component is characterized by UPS-like morphology. (**C**) The heterologous component displays CSA. Case 2: (**D**) The dedifferentiated component shows non-cohesive polygonal cells with moderate eosinophilic cytoplasm and large pleomorphic nuclei. (**E**) Some multinucleated tumor giant cells can also be observed (blue arrows). Brisk mitotic activity (green circle) with occasional atypical mitotic figures (yellow circles) is noted in the dedifferentiated component. (**F**) High-power magnification reveals large polygonal cells possessing abundant eosinophilic cytoplasm and eccentrically placed nuclei with marked pleomorphism, compatible with RMS. Some tumor cell nuclei display one or more conspicuous nucleoli (red circle). Case 3: (**G**,**H**) The differentiated components show the histological features of (**G**) spindle cell LMS and (**H**) epithelioid LMS. (**I**,**J**) The dedifferentiated component shows scattered pleomorphic tumor cells possessing (**I**) abundant eosinophilic cytoplasm (blue arrows) and (**J**) eccentrically placed nuclei with conspicuous macronucleoli (red circle). Original magnification: (**A**) 20×; (**B**), 40×; (**C**–**E**), 200×; (**F**), 400×; (**G**,**H**) 40×; (**I**,**J**) 200×.

**Figure 3:**
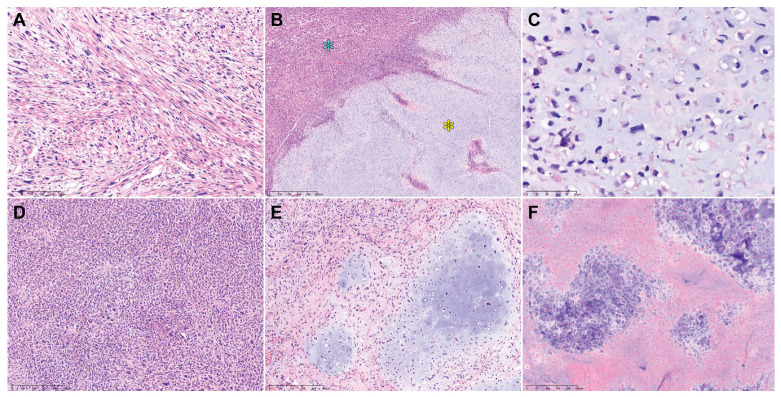
Histological features of uterine DDLMS (cases 4 and 5). Case 4: (**A**) The differentiated area exhibits the classic morphology of malignant smooth muscle differentiation, including intersecting fascicles of atypical spindle cells with eosinophilic cytoplasm (spindle cell LMS). (**B**) The dedifferentiated (blue asterisk) and heterologous (yellow asterisk) components are sharply delineated. (**C**) The heterologous component is morphologically compatible with high-grade CSA. Case 5: (**D**) The dedifferentiated component shows extreme hypercellularity and severe-to-marked nuclear pleomorphism. (**E**) The heterologous component comprises variable-sized islands of malignant cartilage. (**F**) In a few areas showing CSA, extensive tumor necrosis is observed. Original magnification: (**A**) 100×; (**B**) 40×; (**C**) 200×; (**D**–**F**) 100×.

Table 3 summarizes the pathological characteristics of uterine DDLMS. Figure 2 and Figure 3 are photomicrographs showcasing the histological features of case 1 (Figure 2A–C), case 2 (Figure 2D–F), case 3 (Figure 2G–J), case 4 (Figure 3A–C), and case 5 (Figure 3D–F), respectively. Dedifferentiated, undifferentiated, or heterologous morphology was included in the initial pathological diagnosis in all cases. The absence of a benign and malignant epithelial tumor component excluded the possibility of adenosarcoma and carcinosarcoma of endometrial origin. All uterine tumors exhibited varying amounts of LMS types (20–80%), including spindle cell and epithelioid. There was no evidence of co-existing leiomyoma or smooth muscle tumor of uncertain malignant potential (STUMP). The histologically differentiated areas of these tumors demonstrated the morphology typical of malignant smooth muscle differentiation, including intersecting fascicles of atypical spindle cells with eosinophilic cytoplasm, variably well-defined cell borders, broad or blunt-ended nuclei (spindle cell LMS), as well as round or polygonal cells with eosinophilic or clear cytoplasm arranged in nested, corded, nodular, and diffuse patterns (epithelioid LMS). Three tumors (cases 1, 3, and 5) showed mixed spindle cell and epithelioid morphologies, while the remaining two tumors were compatible with epithelioid LMS (case 2) and spindle cell LMS (case 4), respectively. High-grade histological features, including multifocal tumor cell necrosis and brisk mitotic activity, were identified in the differentiated areas of all tumors. The dedifferentiated tumors resembled UPS or malignant fibrous histiocytoma (MFH) of the soft tissue and were characterized by large pleomorphic tumor cells admixed with haphazardly arranged atypical cells with marked nuclear pleomorphism in a background of myxoid or collagenous stroma. The heterologous components, including RMS (case 3) and CSA (case 5), were first detected in the primary uterine tumors. In cases 1, 2, and 4, no dedifferentiated or heterologous component was identified in the primary tumors, but the heterologous components comprised more than half of the metastatic tumor volume. Three cases (cases 1, 4, and 5) showed a sharp demarcation between the differentiated and dedifferentiated components, while in two cases (cases 2 and 3) the dedifferentiated area imperceptibly merged with the differentiated component. The dedifferentiated and heterologous components exhibited an expansile but focally infiltrative, scalloped tumor border; severe nuclear pleomorphism; occasional bizarre, monstrous, or multinucleated tumor cells; and frequent mitoses (range, 21–46 per 10 high-power fields). One case (case 2) contained multinucleated tumor giant cells.

**Figure 4:**
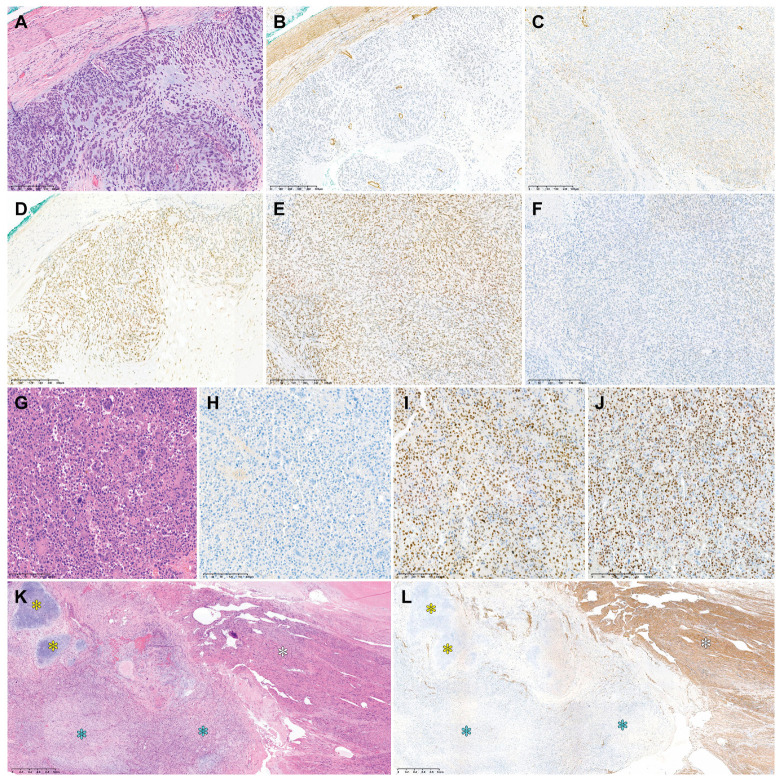
Immunostaining results of uterine DDLMS. Case 1: (**A**) In the dedifferentiated component, desmin immunoreactivity is either (**B**) absent or (**C**) faint in a few tumor cells. The dedifferentiated component shows (**D**) strong S100 protein expression, (**E**) preserved RB protein expression, and (**F**) wild-type p53 protein expression. Case 2: (**G**) The dedifferentiated component resembling UPS shows (**H**) a lack of desmin immunoreactivity, (**I**) strong SATB2 expression, and (**J**) p53 overexpression. Case 4: (**K**) Low-power magnification reveals differentiated LMS (white asterisk), DDLMS (blue asterisk), and CSA (yellow asterisk). (**L**) Desmin expression is uniform and strong in the differentiated component (white asterisk), while the dedifferentiated (blue asterisk) and heterologous (yellow asterisk) components show no signs of desmin expression. Original magnification: (**A**–**F**) 40×; (**G**–**J**) 100×; (**K**,**L**) 20×.

Table 3 summarizes the immunophenotypes of uterine DDLMS, while the photomicrographs in Figure 4 show the immunophenotypical features. Regarding the differentiated component, three cases were diffusely positive for desmin. A few microscopic foci were weakly positive for S100 protein and SATB2 in one case. In all examined cases, myogenin and myoD1 expression was negative, p53 protein expression was wild type, and RB protein expression was preserved. Regarding the dedifferentiated and heterologous components, three cases were completely negative for desmin. In one case, the dedifferentiated area exhibited patchy faint-to-weak staining for desmin. CSA was strongly positive for S100 protein in two cases. Some areas of RMS were weakly positive for myogenin. The dedifferentiated and heterologous components were negative for myoD1 in all cases. One case exhibited diffuse and intense nuclear immunoreactivity for SATB2. p53 protein was overexpressed in one case. RB1 protein expression was preserved in two cases.

## 4. Discussion

DDLMS is defined by the presence of an undifferentiated tumor component, which lacks the histological and immunophenotypical features of smooth muscle differentiation, in proximity with differentiated LMS [20]. DDLMS displays pleomorphic atypical cells with brisk mitotic activity and extensive necrosis, with characteristics similar to UPS. DDLMS of the soft tissue presents as highly aggressive tumors with a 50–65.2% mortality rate and 89% likelihood of metastasis [21,39]. It has also been reported that the loss of myogenic differentiation in LMS could be a significant prognostic factor accounting for the aggressiveness of these tumors [40]. Among soft tissue tumors, dedifferentiation is an infrequent but well-known phenomenon observed in pleomorphic RMS, dedifferentiated chordoma, and dedifferentiated liposarcoma [41]. However, the dedifferentiation of uterine LMS was not mentioned in the most recent World Health Organization Classification of female genital tumors [42].

For a comprehensive review of the literature on uterine DDLMS with a heterologous component, we searched the Medline bibliographic database via the PubMed retrieval service using the keywords “uterus”, “leiomyosarcoma”, “dedifferentiation”, “dedifferentiated leiomyosarcoma”, “heterologous”, “osteosarcoma,” “chondrosarcoma”, and “rhabdomyosarcoma”. We found that 42 previously published cases of primary uterine LMS with a heterologous component have been reported to date [3,16,20,41,43,44,45,46] and subsequently collected the clinicopathological information of these cases. Table 4 summarizes the clinical characteristics of 42 patients with uterine DDLMS. The mean age at diagnosis was 57.7 years (range, 38–90 years). Only a few individual case reports described the initial symptoms or signs, including pelvic or abdominal pain, vaginal bleeding, hypermenorrhea, and infertility. Imaging findings and clinical impressions were available for only a few individual cases. Imaging studies revealed several variable-sized fibroids or solid heterogeneous masses measuring up to 18.9 cm. Staging information was available for 27 cases, more than half of which were classed as stage I (15/27; 55.6%). Six patients had stage IV disease at the time of initial diagnosis. Most patients underwent surgery, including total hysterectomy, bilateral salpingo-oophorectomy, and myomectomy, and approximately one-third received post-operative chemotherapy or radiation therapy. Three of the six patients with stage IV disease were initially prescribed chemotherapy. Of the 17 patients whose post-treatment recurrence data were available, 13 (76.5%) developed locoregional or metastatic recurrences. Similarly, 16 of the 18 patients with available relevant clinical radiological information experienced distant metastases in the lungs, pleura, liver, peritoneum, brain, bones, heart, and kidneys.

Follow-up information was available for 32 of the 42 previously reported patients with uterine DDLMS. Although most of these tumors were classed as stage I at presentation, 62.5% (20/32) of patients died of the disease, with a mean overall survival of 26.7 months. Brief clinical presentations of patients described in each of the previously published articles are as follows: Iihara et al. [46] reported in their single case report that the patient received gamma knife radiotherapy for brain metastasis detected 16 months after initial therapy. She died of metastatic disease at 20 months after initial therapy. According to the case series reported by Chen et al. [20], one of the two patients who underwent mass excision died 12 months after initial presentation, and the other patient who underwent hysterectomy was alive without evidence of disease 28 months after initial presentation. In a single case report by Rawish and Fadare [44], despite hysterectomy with adjuvant chemotherapy, the patient developed a large pelvic mass with multiple peritoneal seeding at 6 months after surgery. She underwent debulking surgery and chemotherapy, and was alive with disease 8 months after hysterectomy. Yu and Hornick [3] recently documented a series of 15 DDLMS cases, in which 10 cases were of uterine origin. Eight patients developed distant metastases in the lungs, liver, bone, small bowel, peritoneum, and so on. Among nine patients whose follow-up information was available, six patients died of disease with a mean overall survival of 10.5 months and three patients were alive with disease with a mean overall survival of 32 months. In another recent case series reported by Chapel et al. [16], survival data were available for 19 patients. Twelve patients died of disease with a median disease-specific survival of 14 months (range, 2–73 months). Four patients were alive with disease at 4, 12, 44, and 50 months, and three patients were alive without evidence of disease at 56, 109, and 114 months. Interestingly, only one patient experienced RFS > 24 months. They stated that the proportion of dedifferentiated component and immunoreactivity were not significantly associated with survival. Kousar et al. [47] reported in their single case report that the patient was treated with hysterectomy with adjuvant chemotherapy. Abdominopelvic computed tomography after completing chemotherapy indicated that she developed multiple peritoneal and liver metastases.

Detailed information on the histological types of differentiated and dedifferentiated components was available for all 42 previously published DDLMS cases. Table 5 summarizes their histological characteristics. Although all tumors contained variable epithelioid, spindled, and pleomorphic tumor cells, the dominant morphology of the differentiated component was spindle cell LMS in more than two-thirds of cases (32/42; 76.2%). One case (2.4%) showed dominant epithelioid LMS in the differentiated areas. Cellular leiomyoma, leiomyoma with bizarre nuclei, and STUMP were identified in three (7.1%), one (2.4%), and two (4.8%) cases, respectively. Three cases (7.1%) showed a transition from benign to high-grade poorly differentiated components. Notably, benign components (leiomyoma, cellular leiomyoma, and leiomyoma with bizarre nuclei) co-existed with spindle cell LMS in two cases, while in another case, areas of frank LMS and undifferentiated component were imperceptibly blended into more recognizable smooth muscle (leiomyoma-like) areas. The tumor size ranged between 3.0 and 30.0 cm. In 23 cases (54.8%), the dedifferentiated component showed MFH- or UPS-like morphology, characterized by large non-cohesive polygonal cells possessing moderate-to-abundant eosinophilic to amphophilic cytoplasm, large pleomorphic nuclei with coarse vesicular-to-smudged chromatin, and one or more macronucleoli. The proportion of dedifferentiated component ranged between 5% and 70%. Although we did not include a case of uterine DDLMS showing an OSA component in this study, our comprehensive review of the previous literature revealed that 13 tumors (31.0%) had areas of OSA as a heterologous component. Particularly, Yu and Hornick [3] reported a series of 10 uterine DDLMS cases showing an OSA component. Some of those tumors also included areas of CSA or RMS.

In their study of 18 DDLMS cases, Chen et al. [7] stated that the delineation of pleomorphic LMS and DDLMS can be challenging, especially in cases where an abrupt morphological transition occurs with no observable shift in immunophenotype. Pleomorphic LMS and DDLMS may represent a histological spectrum of LMS transitioning from classic LMS to high-grade UPS. One of the cases included in their study showed faint-to-weak desmin immunoreactivity in a few microscopic areas of the dedifferentiated component. However, those areas comprised less than 1% of the entire tumor volume, and most tumor tissues did not express desmin. Since the histological features of the dedifferentiated and heterologous components corresponded to UPS and CSA, respectively, a diagnosis of DDLMS was established.

LMS encompasses tumors that demonstrate a wide range of differentiation with loss of smooth muscle marker expression, extending from well-differentiated to poorly differentiated LMS, resembling UPS [48]. The vast majority of patients with uterine LMS carry at least one mutation in either tumor protein 53 (*TP53*), retinoblastoma (*RB1*), phosphatase and tensin homolog deleted on chromosome 10 (*PTEN*), or alpha-thalassemia/mental retardation, X-linked (*ATRX*) [49]. It has been also documented that a small subset of uterine LMS harbors somatic mutations in the breast cancer gene (*BRCA*) and alterations in homologous recombination repair genes [50]. Guo et al. [51] identified three molecular subtypes of LMS and confirmed their findings by analyzing The Cancer Genome Atlas (TCGA) datasets. Subtype III almost entirely corresponded with uterine LMS, with 92% of subtype III samples derived from the uterus [48]. Genes enriched in subtype III involved biological processes regulating transcription and the metabolic pathways and indicated that uterine LMS comprises a molecularly and clinically distinct cluster from soft tissue LMS (subtype I), which frequently overexpresses genes associated with normal smooth muscle function and differentiation [51]. In contrast, subtype II showed significantly fewer muscle-specific genes than other subtypes and indicated a more dedifferentiated molecular subtype [48]. Importantly, this dedifferentiated subtype was shown to cluster with UPS and characterized by significantly higher genomic instability and worse outcome. We consider that these genomically distinct subtypes can partially explain the significant phenotypic and morphological differences between conventional LMS and DDLMS of the uterus.

The phenomenon of cellular plasticity, the ability of cells to change their phenotype in a reversible fashion, is involved in tissue regeneration as well as epithelial homeostasis [52,53]. Cellular plasticity also plays an important role in tumor development and progression [54], and it is closely related to intratumoral heterogeneity and variable degrees of phenotypic interconversion [55]. It is not surprising that malignant tumors present with phenotypes and molecular features of either retrodifferentiated, dedifferentiated, or transdifferentiated states, suggestive of cellular plasticity. Among these, retrodifferentiation and dedifferentiation are often used interchangeably [56]. Retrodifferentiation is characterized by a reversion of maturated properties and expression patterns of a differentiated phenotype to a precursor or stem-like cell [57]. Cancer stem cells (CSCs), which are generated by retrodifferentiation from differentiated tumor cells, regain the capacity for self-renewal and may thus be able to maintain tumorigenicity [58]. Accumulating evidence suggests that certain tumor cells can adopt a CSC state associated with the epithelial–mesenchymal transition, higher transdifferentiation potential, and increased resistance to chemotherapy or radiation therapy [59,60]. While retrodifferentiation implies the acquisition of a stem cell/progenitor phenotype, dedifferentiation of tumor cells is characterized by loss of phenotypic specialization, i.e., morphological loss of lineage identity and expression patterns with tumor progression [61,62]. Some cases of conventional LMS with pleomorphic foci retain smooth muscle marker expression, while others show undifferentiated morphology and no or at most very focal smooth muscle marker immunoreactivity. It has been suggested that the latter might be most rigorously regarded as DDLMS [16,39] and that the former might be categorized as pleomorphic LMS. In this study, all except one examined case was negative for desmin, and the remaining case exhibited only rare cells faintly expressing desmin.

There have been some case reports describing loss and gain of certain morphologies and immunophenotypes in the process of dedifferentiation [63,64,65]. In a case of intracranial anaplastic hemangiopericytoma reported by Tan et al. [65], the dedifferentiated component displayed a focal area of glandular formation with the acquisition of epithelial immunophenotypes. Watts et al. [63] described a rare case of succinate dehydrogenase (SDH)-deficient dedifferentiated gastrointestinal stromal tumor (GIST) of the stomach, exhibiting loss of SDH subunit A expression and gain of the smooth muscle immunophenotype. Dedifferentiation of GIST is a rare but well-recognized phenomenon [66]. It is characterized by transition to a frankly sarcomatoid morphology and frequently accompanied by loss of immunoreactivity for KIT and discovered for GIST 1. In a recent case report written by Shah et al. [64], the patient who was initially diagnosed as having grade 1 endometrioid carcinoma of the endometrium developed an isolated breast metastasis. The tumor underwent dedifferentiation to undifferentiated carcinoma at the metastatic site. Particularly, the metastatic lesion showed undifferentiated morphology and loss of PAX8 expression, without a residual low-grade component. It also demonstrated loss of immunoreactivities for AT-rich interaction domain 1A (ARID1A) and ARID1B, which was not observed in the primary endometrial tumor. To the best of our knowledge, the alteration in stem cell/progenitor phenotype or the acquisition of certain immunophenotypes has never been investigated in uterine DDLMS. Although a recent genomic database analysis by Astolfi et al. [49] revealed that *PTEN* mutation was more frequent in metastatic uterine LMS than in primary ones, this finding was not exactly about DDLMS. It is unclear which genetic and molecular differences may contribute to the distinction between uterine LMS and DDLMS. In order to gain insight about their differences and enlighten differential management, further investigations are warranted to reveal the distinct expression profiles and mutation patterns between conventional LMS and DDLMS of the uterus.

This study has several limitations. First, since we enrolled patients with uterine DDLMS who underwent surgery at a single institution, the cohort was relatively small. Second, comparative molecular analysis was beyond the scope of this study. Third, due to the small number of cases, we did not analyze the statistical differences in survival. Further investigations using more detailed prognostic information obtained from larger cohorts of uterine DDLMS are necessary. Fourth, the divergent criteria used to define DDLMS in previous studies were noted, although we have addressed this through a fair and rational approach, as discussed above.

In conclusion, we demonstrated that dedifferentiation occurs in primary uterine LMS or in recurrent or metastatic tumors. Our findings suggest that a subset of uterine LMS represents various types and amounts of dedifferentiation. Routine prospective recognition of DDLMS and distinction from its mimickers are advocated for accurate diagnosis and further characterization of these rare tumors.

## Figures and Tables

**Table 1:** Antibodies used.

Antibody	Dilution	Clone	Company	Positive Control
Desmin	1:200	D33	Agilent Technologies, Santa Clara, CA, USA	Normal myometrium
S100 protein	1:5000	polyclonal	Agilent Technologies, Santa Clara, CA, USA	Vulvar malignant melanoma
Myogenin	1:50	F5D	Cell marque, Rocklin, CA, USA	Uterine rhabdomyosarcoma
MyoD1	1:50	EP212	Cell marque, Rocklin, CA, USA	Uterine rhabdomyosarcoma
SATB2	1:2000	EPNCIR130A	Abcam, Cambridge, UK	Colonic adenocarcinoma
p53	1:800	DO-7	Leica Biosystems, Deer Park, IL, USA	Ovarian HGSC
Retinoblastoma protein	1:400	4H1	Cell Signaling Technology, Beverly, MA, USA	Ocular retinoblastoma

Abbreviations: HGSC—high-grade serous carcinoma; SATB2—special AT-rich sequence-binding protein 2.

**Table 2:** Clinical characteristics of five patients with uterine DDLMS.

Case No	1	2	3	4	5
Age (years)	60	55	51	51	63
Presenting symptoms	Abdominal discomfort, constipation	Uterine mass detected on routine examination	Uterine masses detected on routine examination	Uterine mass detected on routine examination	Abdominal discomfort and pain, palpable pelvic mass
Previous medical history	None	None	Thyroid cancer, umbilical hernia	Hyperthyroidism	None
Imaging findings of uterine tumor	NA	A large solid cystic mass	Multiple solid uterine masses measuring up to 8.9 cm	An 8.3 cm lobulated, heterogeneous mass with hypodense necrotic area	A 23.0 cm large heterogeneous mass with adnexal extension
Clinical impression	Uterine leiomyoma	Uterine leiomyoma	Uterine leiomyoma	Uterine leiomyoma	Uterine sarcoma
Surgical procedure for uterine tumor	TH	TH, BSO, PLND, PALND, OMT, APP	TH, bilateral salpingectomy	TH, BSO, OMT	Uterine mass excision, LO, LHC, left nephrectomy
Pathological diagnosis of uterine tumor	LMS	DDLMS	DDLMS-H	LMS	DDLMS-H
Greatest dimension of uterine tumor (cm)	NA	27.8	8.9	8.3	23.0
Adnexal extension	Absent	Absent	Absent	Absent	Present
Pelvic extension	Absent	Absent	Present	Absent	Present
Abdominal extension	Absent	Absent	Absent	Absent	Present
LN metastasis	Absent	Absent	Present (para-aortic)	Present (left pelvic)	Absent
Initial stage	IB	IB	IIIC	IIIC	IVC
Post-operative treatment	CCRT	Chemotherapy	RTx (interrupted)	Chemotherapy	NA (lost to follow-up)
Post-treatment recurrence	Present (ovary, colon, bladder, peritoneum, mesentery, omentum)	Present (vagina, bladder, peritoneum, lungs)	Absent	Present (left paracolic gutter)	NA (lost to follow-up)
Pathological diagnosis of recurrent tumor	Metastatic DDLMS-H	Metastatic DDLMS-H	None	Metastatic DDLMS-H	NA (lost to follow-up)
Treatment for recurrence	Excision, RTx, chemotherapy	Excision, chemotherapy	None	Excision	NA (lost to follow-up)
RFS (months)	30	6	15	4	NA (lost to follow-up)
Survival status	Alive with disease	NA (lost to follow-up)	No evidence of disease	NA (lost to follow-up)	NA (lost to follow-up)
OS (months)	93	NA (lost to follow-up)	15	NA (lost to follow-up)	NA (lost to follow-up)

Abbreviations: APP—appendectomy; BSO—bilateral salpingo-oophorectomy; CCRT—concurrent chemoradiation therapy; DDLMS-H—dedifferentiated leiomyosarcoma with heterologous component; LHC—left hemicolectomy; LO—left oophorectomy; NA—not applicable; OMT—omentectomy; RTx—radiation therapy; PALND—para-aortic lymph node dissection; PLND—pelvic lymph node dissection; TH—total hysterectomy.

**Table 3:** Pathological characteristics of five patients with uterine DDLMS.

Case No		1	2	3	4	5
Dominant morphology of differentiated component		Spindle cell and epithelioid LMS	Epithelioid LMS	Spindle cell and epithelioid LMS	Spindle cell LMS	Spindle cell and epithelioid LMS
Nuclear pleomorphism		Severe	Severe	Severe	Severe	Severe
Mitotic count per 10 HPFs		23	40	46	22	21
Tumor cell necrosis		Present	Present	Present	Present	Present
Tumor border		Infiltrative	Infiltrative	Infiltrative	Infiltrative	Infiltrative
Lymphocytic infiltrate		Absent	Present	Present	Present	Absent
Histological type and proportion of heterologous component		CSA (80%)	RMS (60%)	RMS (20%)	CSA (60%)	CSA (30%)
Desmin	Differentiated	DMP	DSP	DSP	Negative	NA
	Dedifferentiated	FWP	Negative	Negative	Negative	NA
S100 protein	Differentiated	FWP	Negative	NA	Negative	NA
	Dedifferentiated	DSP	Negative	NA	FSP	NA
Myogenin	Differentiated	Negative	Negative	Negative	NA	NA
	Dedifferentiated	Negative	Negative	FWP	NA	NA
MyoD1	Differentiated	Negative	Negative	Negative	NA	NA
	Dedifferentiated	Negative	Negative	Negative	NA	NA
SATB2	Differentiated	FWP	Negative	NA	NA	NA
	Dedifferentiated	Negative	DSP	NA	NA	NA
p53	Differentiated	WT	WT	WT	NA	NA
	Dedifferentiated	WT	OE	WT	NA	NA
RB protein	Differentiated	No loss	No loss	NA	NA	NA
	Dedifferentiated	No loss	No loss	NA	NA	NA

Abbreviations: CSA—chondrosarcoma; DMP—diffuse moderate positive; DSP—diffuse strong positive; FSP—focal strong positive; FWP—focal weak positive; HPFs—high-power fields; NA—not applicable; OE—overexpression; RMS—rhabdomyosarcoma; WT—wild type.

**Table 4:** Clinical characteristics of 42 previously reported uterine DDLMS cases.

Authors (Year Published)	Case No	Age (Years)	Presenting Symptom	Initial Stage	Treatment	Recurrence	Distant Metastasis	DFS (Months)	Outcome	OS or DSS (Months)
Iihara et al., (2007) [46]	1	48	Hypermenorrhea for 4 years	IB	UAE, TH, CTx, GKRS	NA	Present (lungs, brain)	NA	DOD	20 ^a^
Chen et al., (2011) [20]	2	59	Pelvic pain	NA	TH, BSO	Absent	Absent	28	NED	28 ^a^
	3	80	PMB	NA	Excision	NA	NA	NA	DOC	12 ^a^
Rawish and Fadare (2012) [44]	4	48	NA	IA	SH, BSO, CTx	Present	Absent	6	AWD	8 ^a^
Parikh et al., (2015) [45]	5	60	Abdominal pain for 4 months	NA	TH, BS	NA	NA	NA	NA	NA
	6	38	Infertility	NA	Myomectomy	NA	NA	NA	NA	NA
Nosaka et al., (2016) [41]	7	63	Bloody vaginal discharge for 2 months	IVB	TH, BSO, CTx	NA	Present (lungs)	NA	NA	NA
Yu and Hornick (2022) [3]	8	63	NA	NA	Surgery, CTx	Present	Present (lungs, diaphragm, heart, SVC, kidney)	NA	DOD	7.5 ^a^
	9	40	NA	NA	Surgery, CTx	Present	Present (colon, retroperitoneum)	NA	DOD	18 ^a^
	10	48	NA	NA	Surgery, CTx, RTx	Present	Present (lungs, liver, peritoneum, colon, small bowel)	NA	DOD	6.5 ^a^
	11	55	NA	NA	CTx	Absent	Present (pancreas, bone, lungs)	NA	DOD	NA
	12	52	NA	NA	Surgery, CTx	Present	Present (lungs, abdomen)	NA	AWD	36 ^a^
	13	61	NA	NA	Surgery, CTx, RTx	Present	Present (lungs, pleura)	NA	DOD	17 ^a^
	14	53	NA	NA	Surgery	Absent	NA	NA	DOD	3.5 ^a^
	15	62	NA	NA	NA	NA	NA	NA	NA	NA
	16	43	NA	NA	Surgery, CTx	Present	Present (small bowel)	NA	AWD	32 ^a^
	17	52	NA	NA	Surgery, CTx	Present	Present (liver, small bowel)	NA	AWD	28 ^a^
Sadiq and Khan (2022) [43]	18	68	Vaginal bleeding	NA	TH, BSO	NA	NA	NA	NA	NA
Chapel et al., (2023) [16]	19	88	NA	IB	Surgery	NA	NA	2	DOD	2 ^b^
	20	66	NA	IB	Surgery	NA	NA	7	DOD	11 ^b^
	21	68	NA	IB	Surgery, CTx	Present	NA	3	DOD	20 ^b^
	22	42	NA	IB	Surgery	NA	NA	NA	DOD	23 ^b^
	23	61	NA	IB	Surgery	NA	NA	NA	DOD	5 ^b^
	24	57	NA	IIIB	Surgery	NA	NA	NA	NA	NA
	25	46	NA	II	Surgery	NA	NA	23	DOD	31 ^b^
	26	54	NA	IB	Surgery	NA	NA	NA	DOD	73 ^b^
	27	59	NA	IB	CTx	NA	NA	114	NED	114 ^b^
	28	50	NA	IIIB	Surgery	NA	NA	12	AWD	12 ^b^
	29	53	NA	IA	Surgery	NA	NA	21	AWD	50 ^b^
	30	63	NA	IV	CTx	NA	Present	8	DOD	16 ^b^
	31	53	NA	IV	CTx	NA	Present	NA	DOD	15 ^b^
	32	54	NA	IV	Surgery	NA	Present	NA	DOD	3 ^b^
	33	52	NA	IV	CTx	NA	Present	NA	DOD	10 ^b^
	34	57	NA	IB	CTx for recurrence	Present	Present	4	NED	109 ^b^
	35	90	NA	II	Surgery	NA	NA	NA	NA	NA
	36	54	NA	II	CTx and RTx for recurrence	Present	NA	2	NED	56 ^b^
	37	70	NA	IB	RTx	NA	NA	15	AWD	44 ^b^
	38	75	NA	IB	Surgery	NA	NA	NA	NA	NA
	39	55	NA	IB	CTx for recurrence	Present	NA	4	AWD	4 ^b^
	40	60	NA	IB	CTx for recurrence	Present	NA	8	DOD	13 ^b^
	41	63	NA	II	Surgery	NA	NA	NA	NA	NA
Kousar et al., (2023) [47]	42	42	Acute abdominal pain and vaginal bleeding	IV	Surgery, CTx	Present	Present (liver, peritoneum)	NA	NA	NA

Abbreviations: AWD—alive with disease; BSO—bilateral salpingo-oophorectomy; CTx—chemotherapy; DFS—disease-free survival; DSS—disease-specific survival; DOD—died of disease; GKRS—gamma knife radiosurgery; NA—not applicable; NED—no evidence of disease; OS—overall survival; PMB—postmenopausal bleeding; SH—subtotal hysterectomy; SVC—superior vena cava; TH—total hysterectomy; UAE—uterine artery embolization. ^a^ OS; ^b^ DSS.

**Table 5:** Pathological characteristics of 42 previously reported uterine DDLMS cases.

Authors (Year Published)	Case No	Tumor Size (cm)	Dominant Morphology of Differentiated Component	Histological Type of Heterologous Component (Proportion)
Iihara et al., (2007) [46]	1	14.0	Spindle cell LMS	Mixed proliferation of scattered bizarre cells and spindle cells without specific structures (NA)
Chen et al., (2011) [20]	2	6.8	Spindle cell LMS	MFH-like morphology (NA)
	3	18.0	Spindle cell LMS	MFH-like morphology (NA)
Rawish and Fadare (2012) [44]	4	18.0	Spindle cell LMS	OSA (10%)
Parikh et al., (2015) [45]	5	30.0	Spindle cell LMS	Osteochondroid differentiation and MNGCs (NA)
	6	12.5	Spindle cell LMS	OSA (NA)
Nosaka et al., (2016) [41]	7	14.0	Spindle cell LMS	Undifferentiated pleomorphic sarcoma (NA)
Yu and Hornick (2022) [3]	8	11.7	Spindle cell LMS	OSA (NA)
	9	NA	Spindle cell LMS	OSA (NA)
	10	12.0	Spindle cell LMS	OSA (NA)
	11	9.0	Spindle cell LMS	OSA (NA)
	12	20.0	Spindle cell LMS	OSA (NA)
	13	18.0	Spindle cell LMS	OSA (NA)
	14	16.0	Spindle cell LMS	OSA (NA)
	15	18.0	Spindle cell LMS	OSA (NA)
	16	17.0	Spindle cell LMS	OSA (NA)
	17	18.8	Spindle cell LMS	OSA (NA)
Sadiq and Khan (2022) [43]	18	Up to 4.0	Spindle cell LMS	CSA (NA)
Chapel et al., (2023) [16]	19	NA	Spindle cell LMS	MFH-like morphology (30%)
	20	6.5	Spindle cell LMS	MFH-like morphology (20%)
	21	8.5	Spindle cell LMS and CLM	MFH-like morphology (40%)
	22	10.0	CLM	MFH-like morphology (50%)
	23	7.0	Epithelioid LMS	MFH-like morphology (5%)
	24	12.0	Spindle cell LMS	MFH-like morphology (15%)
	25	12.1	Spindle cell LMS	MFH-like morphology and OSA (20%)
	26	8.2	Spindle cell LMS	MFH-like morphology (10%)
	27	6.8	Spindle cell LMS	MFH-like morphology (40%)
	28	3.0	Spindle cell LMS	MFH-like morphology (50%)
	29	4.5	STUMP	MFH-like morphology (30%)
	30	11.7	Spindle cell LMS	MFH-like morphology and OSA (10%)
	31	8.0	Spindle cell LMS	MFH-like morphology (25%)
	32	25.0	Spindle cell LMS	MFH-like morphology (20%)
	33	20.0	Spindle cell LMS	MFH-like morphology, OSA, and CSA (70%)
	34	9.0	CLM	MFH-like morphology (50%)
	35	20.0	LBN	MFH-like morphology (50%)
	36	14.5	CLM	MFH-like morphology (20%)
	37	11.0	STUMP	MFH-like morphology (40%)
	38	6.1	Spindle cell LMS	MFH-like morphology (70%)
	39	10.0	Spindle cell LMS	MFH-like morphology (40%)
	40	14.3	LM, LBM, and spindle cell LMS	MFH-like morphology (10%)
	41	7.7	Spindle cell LMS	MFH-like morphology (70%)
Kousar et al., (2023) [47]	42	10.5, 6.7 (fundus); 1.7 (body)	LM-like areas, STUMP, and frank LMS	Areas showing extreme hypercellularity and large polygonal cells with significant cytological atypia and brisk mitotic activity

Abbreviations: CLM—cellular leiomyoma; LBN—leiomyoma with bizarre nuclei; LM—leiomyoma; MFH—malignant fibrous histiocytoma; MNGCs—multinucleated giant cells; NA—not applicable; STUMP—smooth muscle tumor of uncertain malignant potential.

## Data Availability

Data is contained within the article.

## References

[1] Asano H., Isoe T., Ito Y.M., Nishimoto N., Watanabe Y., Yokoshiki S., Watari H. (2022). Status of the current treatment options and potential future targets in uterine leiomyosarcoma: A Review. Cancers.

[2] Devaud N., Vornicova O., Abdul Razak A.R., Khalili K., Demicco E.G., Mitric C., Bernardini M.Q., Gladdy R.A. (2022). Leiomyosarcoma: Current clinical management and future horizons. Surg. Oncol. Clin. N. Am..

[3] Yu S., Hornick J.L. (2022). Malignant mesenchymoma revisited: A clinicopathologic study of leiomyosarcomas with osteosarcomatous differentiation. Am. J. Surg. Pathol..

[4] Penel N., Coindre J.M., Giraud A., Terrier P., Ranchere-Vince D., Collin F., Guellec S.L.E., Bazille C., Lae M., de Pinieux G. (2018). Presentation and outcome of frequent and rare sarcoma histologic subtypes: A study of 10,262 patients with localized visceral/soft tissue sarcoma managed in reference centers. Cancer.

[5] Gronchi A., Strauss D.C., Miceli R., Bonvalot S., Swallow C.J., Hohenberger P., Van Coevorden F., Rutkowski P., Callegaro D., Hayes A.J. (2016). Variability in patterns of recurrence after resection of primary retroperitoneal sarcoma (RPS): A report on 1007 patients from the multi-institutional collaborative RPS Working Group. Ann. Surg..

[6] Buzinskiene D., Mikenas S., Drasutiene G., Mongirdas M. (2018). Uterine sarcoma: A clinical case and a literature review. Acta Med. Litu..

[7] Abeler V.M., Royne O., Thoresen S., Danielsen H.E., Nesland J.M., Kristensen G.B. (2009). Uterine sarcomas in Norway. A histopathological and prognostic survey of a total population from 1970 to 2000 including 419 patients. Histopathology.

[8] D’Angelo E., Prat J. (2010). Uterine sarcomas: A review. Gynecol. Oncol..

[9] Hosh M., Antar S., Nazzal A., Warda M., Gibreel A., Refky B. (2016). Uterine sarcoma: Analysis of 13,089 cases based on Surveillance, Epidemiology, and End Results Database. Int. J. Gynecol. Cancer.

[10] Toro J.R., Travis L.B., Wu H.J., Zhu K., Fletcher C.D., Devesa S.S. (2006). Incidence patterns of soft tissue sarcomas, regardless of primary site, in the Surveillance, Epidemiology and End Results Program, 1978–2001: An analysis of 26,758 cases. Int. J. Cancer.

[11] Sparic R., Andjic M., Babovic I., Nejkovic L., Mitrovic M., Stulic J., Pupovac M., Tinelli A. (2022). Molecular insights in uterine leiomyosarcoma: A systematic review. Int. J. Mol. Sci..

[12] Roberts M.E., Aynardi J.T., Chu C.S. (2018). Uterine leiomyosarcoma: A review of the literature and update on management options. Gynecol. Oncol..

[13] Juhasz-Boss I., Gabriel L., Bohle R.M., Horn L.C., Solomayer E.F., Breitbach G.P. (2018). Uterine leiomyosarcoma. Oncol. Res. Treat..

[14] Zhang F., Liu Y., Quan Q., Meng Y., Mu X. (2021). Diagnostic value of preoperative CA125, LDH and HE4 for leiomyosarcoma of the female reproductive system. Cancer Manag. Res..

[15] Henricks W.H., Chu Y.C., Goldblum J.R., Weiss S.W. (1997). Dedifferentiated liposarcoma: A clinicopathological analysis of 155 cases with a proposal for an expanded definition of dedifferentiation. Am. J. Surg. Pathol..

[16] Chapel D.B., Maccio L., Bragantini E., Zannoni G.F., Quade B.J., Parra-Herran C., Nucci M.R. (2023). Dedifferentiated leiomyosarcoma of the uterus: A clinicopathologic and immunohistochemical analysis of 23 cases. Histopathology.

[17] Shmookler B.M., Lauer D.H. (1983). Retroperitoneal leiomyosarcoma. A clinicopathologic analysis of 36 cases. Am. J. Surg. Pathol..

[18] Dekanic A., Jonjic N., Savic Vukovic A. (2022). Dedifferentiated leiomyosarcoma of the auricle with heterologous osteosarcoma component: Case report and literature review. Case Rep. Otolaryngol..

[19] Gaeta R., Matera D., Muratori F., Roselli G., Baldi G., Campanacci D.A., Franchi A. (2020). Dedifferentiated soft tissue leiomyosarcoma with heterologous osteosarcoma component: Case report and review of the literature. Clin. Sarcoma Res..

[20] Chen E., O’Connell F., Fletcher C.D. (2011). Dedifferentiated leiomyosarcoma: Clinicopathological analysis of 18 cases. Histopathology.

[21] Nicolas M.M., Tamboli P., Gomez J.A., Czerniak B.A. (2010). Pleomorphic and dedifferentiated leiomyosarcoma: Clinicopathologic and immunohistochemical study of 41 cases. Hum. Pathol..

[22] Matsuda K., Akazawa Y., Yamaguchi Y., Mussazhanova Z., Kurohama H., Ueki N., Kohno M., Fukushima A., Kajimura I., Hiraki H. (2019). Immunofluorescence analysis of DNA damage response protein p53-binding protein 1 in a case of uterine dedifferentiated leiomyosarcoma arising from leiomyoma. Pathol. Res. Pract..

[23] Zuo X., Wu W.L., Shi P., Liu T.M., Yu N., Li L. (2023). A case report of recurrent leiomyosarcoma with chondrosarcoma differentiation in the abdominal wall and a review of the literature. Pathol. Oncol. Res..

[24] Park S., Park E., Kim H.S. (2022). Mesonephric-like carcinosarcoma of the uterine corpus: Clinicopathological, molecular and prognostic characteristics in comparison with uterine mesonephric-like adenocarcinoma and conventional endometrial carcinosarcoma. Cancer Genom. Proteom..

[25] Chu J., Yeo M.K., Lee S.H., Lee M.Y., Chae S.W., Kim H.S., Do S.I. (2022). Clinicopathological and prognostic significance of programmed death ligand-1 SP142 expression in 132 patients with triple-negative breast cancer. In Vivo.

[26] Sohn J., Lee Y., Kim H.S. (2022). Endometrioid carcinomas of the ovaries and endometrium involving endocervical polyps: Comprehensive clinicopathological analyses. Diagnostics.

[27] Chung Y., Kim S., Kim H.S., Do S.I. (2022). High receptor-interacting serine/threonine-protein kinase 3 (RIP3) expression serves as an independent poor prognostic factor for triple-negative breast carcinoma. Anticancer. Res..

[28] Koh H.H., Park E., Kim H.S. (2022). Mesonephric-like adenocarcinoma of the ovary: Clinicopathological and molecular characteristics. Diagnostics.

[29] Yoo H., Kim H.S. (2023). Clinicopathological and prognostic values of telomerase reverse transcriptase (*TERT*) promoter mutations in ovarian clear cell carcinoma for predicting tumor recurrence, platinum resistance and survival. Cancer Genom. Proteom..

[30] Park S., Cho Y., Kim H.S. (2023). Mesonephric-like adenocarcinoma of the uterine corpus: Clinicopathological and prognostic significance of L1 cell adhesion molecule (L1CAM) over-expression. Anticancer. Res..

[31] Koh H.H., Park E., Kim H.S. (2023). Mesonephric-like adenocarcinoma of the uterine corpus: Genomic and immunohistochemical profiling with comprehensive clinicopathological analysis of 17 consecutive cases from a single institution. Biomedicines.

[32] Kim H., Kim H.S. (2023). Mesonephric-like adenocarcinoma of the uterine corpus: Comparison between mismatch repair protein immunostaining and microsatellite instability testing. Anticancer Res..

[33] Lee Y., Oh Y.L. (2023). Thyroid pathology, a clue to *PTEN* hamartoma tumor syndrome. J. Pathol. Transl. Med..

[34] Park S., Kim J., Jang W., Kim K.M., Jang K.T. (2023). Clinicopathologic significance of the delta-like ligand 4, vascular endothelial growth factor, and hypoxia-inducible factor-2alpha in gallbladder cancer. J. Pathol. Transl. Med..

[35] Chang S., Choi Y.L., Shim H.S., Lee G.K., Ha S.Y., Korean Cardiopulmonary Pathology Study Group (2022). Usefulness of BRAF VE1 immunohistochemistry in non-small cell lung cancers: A multi-institutional study by 15 pathologists in Korea. J. Pathol. Transl. Med..

[36] Koh H.H., Oh Y.L. (2022). Papillary and medullary thyroid carcinomas coexisting in the same lobe, first suspected based on fine-needle aspiration cytology: A case report. J. Pathol. Transl. Med..

[37] Kim H., Na K., Bae G.E., Kim H.S. (2021). Mesonephric-like adenocarcinoma of the uterine corpus: Comprehensive immunohistochemical analyses using markers for mesonephric, endometrioid and serous tumors. Diagnostics.

[38] Kobel M., Ronnett B.M., Singh N., Soslow R.A., Gilks C.B., McCluggage W.G. (2019). Interpretation of p53 immunohistochemistry in endometrial carcinomas: Toward increased reproducibility. Int. J. Gynecol. Pathol..

[39] Oda Y., Miyajima K., Kawaguchi K., Tamiya S., Oshiro Y., Hachitanda Y., Oya M., Iwamoto Y., Tsuneyoshi M. (2001). Pleomorphic leiomyosarcoma: Clinicopathologic and immunohistochemical study with special emphasis on its distinction from ordinary leiomyosarcoma and malignant fibrous histiocytoma. Am. J. Surg. Pathol..

[40] Demicco E.G., Boland G.M., Brewer Savannah K.J., Lusby K., Young E.D., Ingram D., Watson K.L., Bailey M., Guo X., Hornick J.L. (2015). Progressive loss of myogenic differentiation in leiomyosarcoma has prognostic value. Histopathology.

[41] Nosaka K., Komatsu H., Oishi T., Horie Y., Harada T., Umekita Y. (2016). A Case of dedifferentiated leiomyosarcoma of the uterus. Int. J. Pathol. Clin. Res..

[42] WHO, WHO Classification of Tumors Editorial Board (2020). WHO Classification of Tumours: Female Genital Tumours.

[43] Sadiq Q., Khan F. (2022). High grade sarcoma with chondrosarcomatous differentiation in primary uterine leiomyosarcoma; A rare case and review of literature. Gynecol. Oncol. Rep..

[44] Rawish K.R., Fadare O. (2012). Dedifferentiated leiomyosarcoma of the uterus with heterologous elements: A potential diagnostic pitfall. Case Rep. Obstet. Gynecol..

[45] Parikh P., Maheshwari A., Rekhi B. (2015). Two uncommon cases of uterine leiomyosarcomas displaying heterologous osteosarcomatous de-differentiation. J. Cancer Res. Ther..

[46] Iihara K., Hirano K., Fujioka Y., Sakamoto A. (2007). Leiomyosarcoma with dedifferentiation in a premenopausal patient discovered after uterine artery embolization. Pathol. Int..

[47] Kousar A., Wald A.I., Heayn M., Cardillo N.D., Elishaev E., Bhargava R. (2023). Dedifferentiated leiomyosarcoma: Morphology, immunohistochemistry, and molecular findings of a case and review of literature. Int. J. Gynecol. Pathol..

[48] Cope B.M., Traweek R.S., Lazcano R., Keung E.Z., Lazar A.J., Roland C.L., Nassif E.F. (2023). Targeting the molecular and immunologic features of leiomyosarcoma. Cancers.

[49] Astolfi A., Nannini M., Indio V., Schipani A., Rizzo A., Perrone A.M., De Iaco P., Pirini M.G., De Leo A., Urbini M. (2020). Genomic database analysis of uterine leiomyosarcoma mutational profile. Cancers.

[50] Ciccarone F., Bruno M., De Paolis E., Piermattei A., De Bonis M., Lorusso D., Zannoni G.F., Normanno N., Minucci A., Scambia G. (2022). Role of homologous recombination repair (HRR) genes in uterine leiomyosarcomas: A retrospective analysis. Cancers.

[51] Beck A.H., Lee C.H., Witten D.M., Gleason B.C., Edris B., Espinosa I., Zhu S., Li R., Montgomery K.D., Marinelli R.J. (2010). Discovery of molecular subtypes in leiomyosarcoma through integrative molecular profiling. Oncogene.

[52] Hass R., von der Ohe J., Ungefroren H. (2020). The intimate relationship among EMT, MET and TME: A T(ransdifferentiation) E(nhancing) M(ix) to be exploited for therapeutic purposes. Cancers.

[53] Varga J., Greten F.R. (2017). Cell plasticity in epithelial homeostasis and tumorigenesis. Nat. Cell Biol..

[54] Meacham C.E., Morrison S.J. (2013). Tumour heterogeneity and cancer cell plasticity. Nature.

[55] Hammerlindl H., Schaider H. (2018). Tumor cell-intrinsic phenotypic plasticity facilitates adaptive cellular reprogramming driving acquired drug resistance. J. Cell Commun. Signal..

[56] Hass R. (2009). Retrodifferentiation: A mechanism for cellular regeneration?. Biol. Chem..

[57] Hass R. (1994). Retrodifferentiation and cell death. Crit. Rev. Oncog..

[58] Cabillic F., Corlu A. (2016). Regulation of transdifferentiation and retrodifferentiation by inflammatory cytokines in hepatocellular carcinoma. Gastroenterology.

[59] Lu W., Kang Y. (2019). Epithelial-mesenchymal plasticity in cancer progression and metastasis. Dev. Cell.

[60] Easwaran H., Tsai H.C., Baylin S.B. (2014). Cancer epigenetics: Tumor heterogeneity, plasticity of stem-like states, and drug resistance. Mol. Cell.

[61] Yamada Y., Haga H., Yamada Y. (2014). Concise review: Dedifferentiation meets cancer development: Proof of concept for epigenetic cancer. Stem Cells Transl. Med..

[62] Dubois-Pot-Schneider H., Fekir K., Coulouarn C., Glaise D., Aninat C., Jarnouen K., Le Guevel R., Kubo T., Ishida S., Morel F. (2014). Inflammatory cytokines promote the retrodifferentiation of tumor-derived hepatocyte-like cells to progenitor cells. Hepatology.

[63] Watts F., Stewart P., Gill A.J., Krishnaswamy M. (2023). SDHA deficient dedifferentiated gastrointestinal stromal tumour with a smooth-muscle immunophenotype. Pathology.

[64] Shah V.I., Morgan S.E., Kobel M., Lee C.H., McCluggage W.G. (2022). Dedifferentiation in breast metastasis of endometrial carcinoma: A diagnostic dilemma. Int. J. Gynecol. Pathol..

[65] Tan N.J.H., Sun I.S.Y., Low S.W., Kuick C.H., Chang K.T.E., Tan C.L. (2019). A rapidly fatal intracranial anaplastic hemangiopericytoma with de-novo dedifferentiation: Emphasis on diagnostic recognition, molecular confirmation and discussion on treatment dilemma. Brain Tumor Pathol..

[66] Pauwels P., Debiec-Rychter M., Stul M., De Wever I., Van Oosterom A.T., Sciot R. (2005). Changing phenotype of gastrointestinal stromal tumours under imatinib mesylate treatment: A potential diagnostic pitfall. Histopathology.

